# Revisiting Oral Fluoroquinolone and Multivalent Cation Drug-Drug Interactions: Are They Still Relevant?

**DOI:** 10.3390/antibiotics8030108

**Published:** 2019-07-31

**Authors:** Stuart K. Pitman, Uyen T. P. Hoang, Caren H. Wi, Mona Alsheikh, Dakota A. Hiner, Kelly M. Percival

**Affiliations:** 1 College of Pharmacy, University of Iowa, Iowa City, IA 52242, USA; 2 Department of Pharmaceutical Care, University of Iowa Hospitals and Clinics, Iowa City, IA 52242, USA

**Keywords:** drug interaction, fluoroquinolone, cation, oral, antibiotic, resistance

## Abstract

Fluoroquinolones are a widely-prescribed, broad-spectrum class of antibiotics with several oral formulations notable for their high bioavailability. For certain infections, fluoroquinolones are the first line or only treatment choice. When administered orally, fluoroquinolones require proper administration to ensure adequate systemic absorption and, thereby, protect patients from treatment failure. Oral drug preparations that contain multivalent cations are well known to chelate with fluoroquinolones in the gastrointestinal tract; co-administration may lead to clinically significant decreases in oral fluoroquinolone bioavailability and an overall increase in fluoroquinolone-resistant bacteria. Based on a search and evaluation of the literature, this focused review describes oral fluoroquinolone-multivalent cation drug-drug interactions and their magnitude and offers several clinical management strategies for these potentially clinically significant interactions.

## 1. Introduction

Fluoroquinolones may allow practitioners to prescribe an oral treatment for infections that would otherwise require an intravenous agent, possibly avoiding or reducing a patient’s hospital stay. However, the healthcare team—including prescribers, pharmacists, and nurses—must actively ensure proper administration of the oral fluoroquinolone. This includes addressing the frequently encountered and potentially clinically significant drug-drug interactions between oral fluoroquinolones and orally-administered multivalent cations, as these drug interactions may lead to therapeutic failures of the antibiotic and the development of bacterial resistance [1,2,3,4,5,6,7,8].

Oral fluoroquinolone-multivalent cation drug-drug interactions first came into focus in the latter half of the 1980s as detailed in several case reports, drug-drug interaction studies, and reviews. Today, oral fluoroquinolone-multivalent cation interactions are firmly established. However, unawareness among the healthcare team, perhaps due to the passage of time or due to the presence of drug-interaction-electronic-alert fatigue, may allow for many of these interactions to undergo suboptimal review or even persist unnoticed [9]. This concise and focused review describes the oral fluoroquinolone-multivalent cation drug-drug interaction and consolidates relevant data. This review goes beyond the manufacturer-provided package inserts by describing limitations to the manufacturer-recommended interaction management strategy of dose spacing and by proposing additional practical strategies. Available evidence leads to the conclusion that oral fluoroquinolone-multivalent cation drug-drug interactions are still important in many scenarios and deserve the attention of today’s clinician.

The reader should note that this concise review focuses on ciprofloxacin, levofloxacin, moxifloxacin, and delafloxacin but appreciate that other fluoroquinolones significantly interact with multivalent cations as well. Clinically significant interactions between food, juices, non-calcium based phosphate binders (i.e., lanthanum or sevelamer), didanosine, or enteral feeds are not addressed. Lastly, tetracyclines—another antibiotic class prone to chelation-based interactions—are not discussed.

## 2. Background

The spectrum of activity of early fluoroquinolones, such as ciprofloxacin, includes mostly Gram-negative and atypical bacteria [10]. Later fluoroquinolones expanded this spectrum to include some Gram-positive coverage, notably *Streptococcus pneumoniae* (e.g., levofloxacin, moxifloxacin, delafloxacin), methicillin-resistant *Staphylococcus aureus* (delafloxacin), and anaerobes (e.g., moxifloxacin, delafloxacin) [10]. All fluoroquinolones exert their bactericidal activity by targeting bacterial DNA gyrase and topoisomerase IV, which are enzymes necessary for essential bacterial DNA processes [11].

Multivalent cations are present in many frequently used oral drug products, such as antacids that combine weak bases with multivalent cations such as calcium (Ca^2+^), aluminum (Al^3+^) or magnesium (Mg^2+^), calcium supplements such as calcium carbonate, and iron supplements (Fe^3+^) including ferrous sulfate. Sucralfate, a multivalent cation-containing prescription medication, consists of aluminum hydroxide and sulfated sucrose [12].

The oral bioavailability of delafloxacin is 59%, ciprofloxacin 70%, moxifloxacin 89%, and levofloxacin 99% [11,13]. Due to their oral bioavailability, oral doses of fluoroquinolones can be substituted for their intravenous counterparts (at the same dose for moxifloxacin and levofloxacin, and at a higher dose for oral ciprofloxacin and delafloxacin) in many clinical scenarios, assuming no concurrent medications or conditions that would reduce oral absorption. The leading pharmacokinetic-pharmacodynamic indices used to measure fluoroquinolone dosing and response are the area under the concentration-time curve (AUC) to MIC (minimum inhibitory concentration) ratio (AUC:MIC) and peak concentration (Cmax) to MIC ratio (peak:MIC) [10]. Alterations in oral absorption may lead to a decrease in Cmax and reduce the AUC [14]. 

Although the exact mechanism for how oral multivalent cations alter oral fluoroquinolone pharmacokinetics has not been fully elucidated, the leading hypothesis is that a poorly absorbed insoluble complex forms within the gastrointestinal (GI) tract when the multivalent cation chelates with the 4-oxo and adjacent carboxyl groups present on all fluoroquinolone compounds [12,15,16].

Oral ciprofloxacin-multivalent cation interaction studies found alterations in ciprofloxacin absorption pharmacokinetic parameters when administered simultaneously with calcium, aluminum, magnesium, iron, sucralfate, or multivitamins with minerals [12,15,17]. Of these, the magnitude of AUC and Cmax reduction of ciprofloxacin appears greatest with coadministration of aluminum, magnesium, or sucralfate. A lesser impact is present with calcium or iron and is variable with multivitamins depending on their mineral content [17,18] (see Table 1). Readers are referred to an extensive review of the early fluoroquinolone-multivalent cation drug-drug interaction studies for additional information [17].

Prior to the United States’ Food and Drug Administration’s (FDA)-drug approval, randomized crossover absorption studies with oral levofloxacin (100 mg) conducted in healthy men examined coadministration with aluminum hydroxide, magnesium oxide, calcium carbonate, and ferrous sulfate. The pharmacokinetic results are listed in Table 1. Calcium carbonate did not significantly affect the AUC or Cmax (~3% and ~23% respectively), but a 23% decrease in Cmax may be clinically significant in cases where MICs are close to the breakpoint [19]. It is worth noting that the investigators used a non-standard dose of levofloxacin; however, one should expect these drug-drug interactions to persist when using standard doses (e.g., 500 mg) based on fluoroquinolone-multivalent cation pharmacokinetic models, and to yield similar or even greater decreases in the levofloxacin AUC [22].

Prior to FDA-drug approval, oral moxifloxacin (400 mg) was administered as a single dose in several randomized crossover studies in healthy young adult males who co-ingested either sucralfate (190 mg of elemental aluminum), calcium (500 mg of elemental calcium), aluminum in combination with magnesium (900 mg aluminum hydroxide and 600 mg magnesium hydroxide), or ferrous sulfate (100 mg of elemental iron) [20,21] (see Table 1). All interactions were significant with the exception of calcium.

Interaction information with oral delafloxacin and multivalent cations is limited to the manufacturer-provided package insert which states that aluminum, magnesium, sucralfate, iron, or multivitamins with iron or zinc may impair absorption resulting in lower-than-desired systemic delafloxacin concentrations [23].

Oral fluoroquinolone-multivalent cation drug-drug interactions commonly occur in inpatient hospital settings [8,24,25,26,27,28,29,30,31]. Based on extrapolated data from a subset of patients, one hospital estimated that one out of every three levofloxacin doses administered at their institution was given within 2 h of a multivalent cation. However, they included multivitamins, which accounted for approximately 40% of their coadministration. In the absence of iron or zinc, multivitamins have limited data to support causing a significant interaction with levofloxacin [8]. Even with multivitamins removed from their analysis, this still represents a large number of multivalent cation-levofloxacin administrations. A recent retrospective chart review demonstrated that oral fluoroquinolone-multivalent cation drug-drug interactions are also common in hospitalized pediatric patients receiving enteral medications [31].

Clinical failures have been attributed to fluoroquinolone-multivalent cation drug-drug interactions [1,2,3,4,5,6]. Patients systemically infected with organisms having high MICs may be at particular risk [16]. Even a small reduction in fluoroquinolone absorption could be clinically significant in select patients that have “narrow margin[s] for acceptable decreases in bioavailability” [17]. When encountering patients who appear to be failing oral fluoroquinolone therapy, providers should consider whether a drug-drug interaction may have caused or contributed to the patients’ condition. Further, ordering cultures would help differentiate if resistance had emerged or whether the dose may have simply been ineffective due to a fluoroquinolone-multivalent cation drug-drug interaction.

The overall rise in the percentage of fluoroquinolone-resistant bacteria is troublesome, particularly among Gram-negative bacteria [10]. There are concerns that bacterial exposure to fluoroquinolones at lower-than-desired concentrations due to chelation-based interactions may lead to increasing fluoroquinolone resistance by placing the concentration lower in the mutant selection window [7,32]. Avoidance of co-administration of multivalent cations and fluoroquinolones has been posited as a strategy for suppressing the emergence of fluoroquinolone resistance [8,33,34].

## 3. Clinical Management

The healthcare team has several clinical management options when encountering a drug-drug interaction between an oral multivalent cation and an oral fluoroquinolone (Table 2). Before a strategy is chosen, it is imperative to assess the relative magnitude of the interaction between the particular multivalent cation and fluoroquinolone, the clinical situation and setting, the risk versus benefit and feasibility of the proposed approach, the evidence supporting a given strategy, and the availability and appropriateness of alternative agents.

A common recommendation to avoid or minimize fluoroquinolone-multivalent cation drug-drug interactions is to space out the administration times between the drugs to facilitate each drug’s independent absorption by avoiding chelation in the GI tract. This strategy is recommended by the manufacturers (see Table 3). Nix et al. demonstrated that when administering 30 mL of Maalox (17.3 mmol of aluminum ion and 20.6 mmol of magnesium ion) 6 h after the ciprofloxacin dose, no change in relative bioavailability of ciprofloxacin occurred [16]. Other studies have confirmed spacing out administration may eliminate or minimize the interaction between ciprofloxacin and other multivalent cation pairs [17]. Dose spacing for moxifloxacin with multivalent cations has only been studied with the combination of an oral aluminum and magnesium combination product. When 12 healthy males were given moxifloxacin (400 mg) 2 h before, simultaneously, or 4 h after the administration of the combination aluminum-magnesium product, the mean moxifloxacin AUC was reduced 26%, 59%, and 23% respectively. There was no change between the mean Cmax values [21,35]. However, an AUC reduction of 23–26% using a dose spacing strategy for moxifloxacin and multivalent cations could be clinically significant in patients infected with bacteria whose MICs are close to the breakpoints. Dose spacing for oral levofloxacin with multivalent cations has only been studied with sucralfate. Compared to those healthy volunteers who only took a single 500 mg tablet of levofloxacin, the Cmax increased by 13% and the AUC decreased by 5% in those patients who took sucralfate (1 g) 2 h after levofloxacin [36].

It is important to consider the type of evidence that supports a dose spacing strategy. Only a small number of reports describe outcomes of this approach in clinical practice [2,5,6]. Most evaluations of dose spacing were done in young, healthy adults, which limits extrapolation to other populations, such as the ill, elderly, children, or those with delayed gastric emptying (e.g., patients with cystic fibrosis) [16,18,39,40]. Additionally, often single doses of one or both agents were used in those evaluations [39]. Therefore, because of limitations in evidence and for other practical reasons (see Table 2), temporarily discontinuing an interacting multivalent cation during fluoroquinolone use instead of attempting to space them apart is preferred in many cases. 

Another strategy is to substitute a different drug for one of the interacting medications. For example, ranitidine could be substituted for an aluminum-magnesium based antacid, as ranitidine does not interact with ciprofloxacin, levofloxacin, moxifloxacin, or delafloxacin [16,19,21,23]. Or, instead of using a fluoroquinolone, an antibiotic from a different class could be selected.

Switching the oral fluoroquinolone to an intravenous formulation is a less attractive strategy but may be necessary in select cases. This switch could be considered if a patient is treated in an inpatient setting and the multivalent cation cannot be stopped, dose spacing is not feasible or advisable, and an alternative antibiotic cannot be given. 

Various in-hospital programs, often using dose spacing, have attempted to manage oral fluoroquinolone-multivalent cation drug-drug interactions on a larger scale [26,28,29,30,41]. Results are mixed, and when positive results were achieved, it is mostly unknown if those results were maintained over prolonged periods of time [26,28,29]. Lack of prescriber, pharmacist, and nursing awareness of these interactions and their potential consequences and the burdens of multiple administration times throughout the day are possible barriers to successful interventions [30,41]. 

Even with these known barriers, we suggest health systems continue to pursue the use of hospital/systems-wide programs to manage oral fluoroquinolone-multivalent cation drug-drug interactions. Hospital-associated interventions could include standardized education for nurses, pharmacists and physicians; optimization of the electronic medication record and drug-drug interaction software functionality—including developing and using real-time monitoring tools within the electronic medical record; development of collaborative practice protocols that authorize a large number of individuals to participate in fluoroquinolone-multivalent cation drug-drug interaction strategies (see Table 2); and institutional support of research and dissemination of best practices in this area. Lastly, for those inpatient staff involved in procuring and documenting patients’ home medication lists, it is essential to ask about patients’ use of products that contain multivalent cations in order to enable those prescribing fluoroquinolones for home-going use the opportunity to prospectively manage potential interactions. 

## 4. Conclusions

Oral fluoroquinolones are an important tool for the clinician, but they must be administered properly to minimize the chances of therapeutic failure, maximize the chances of clinical cure, and curb the development of resistance. Data dictate that many oral fluoroquinolone-multivalent cation drug-drug interactions remain clinically significant today. When encountering a drug-drug interaction between an oral multivalent cation and an oral fluoroquinolone pair, the health care team must pause and seriously consider this drug-drug interaction, and choose a management strategy appropriate for the particular interaction and patient.

## Figures and Tables

**Table 1:** Reported area under the concentration-time curve (AUC) and peak concentration (Cmax) alterations with co-administered oral fluoroquinolone-multivalent cation pairs [17,19,20,21].

Fluoroquinolone	Cation	Mean Reduction in AUC Compared to Quinolone Alone	Mean Reduction in Cmax Compared to Quinolone Alone
Ciprofloxacin	Aluminum with Magnesium	85%	80%
	Aluminum	85%	81%
	Sucralfate	88% ^a^	90–96% ^a^
	Calcium ^b^	41^c^–42%	38^c^–47%
	Iron Preparations ^d^	42–64%	33–57%
	Multivitamin Preparation Containing Zinc, Magnesium, Calcium, Manganese, Copper, and Iron	52%	53%
	Multivitamin Preparation Containing Zinc and Copper	23%^e^	Not tested
Levofloxacin	Aluminum Hydroxide	44%	65%
	Magnesium Oxide	22%	38%
	Ferrous Sulfate	19%	45%
	Calcium Carbonate	3%	23%
Moxifloxacin	Sucralfate^f^	60%	71%
	Aluminum Hydroxide and Magnesium Hydroxide	59%	61%
	Ferrous sulfate^g^	39%	59%
	Calcium^h^	3%	16%

^a^ Included 1–2 days of pretreatment with sucralfate. ^b^ These percentages do not include calcium acetate which is typically used for phosphorus binding in chronic kidney disease. Concomitant administration of calcium acetate will also significantly decrease the bioavailability of ciprofloxacin. ^c^ Included 6 days of pretreatment with calcium. ^d^ Studies included 0–28 days of pretreatment with iron. ^e^ Included 5 days of pretreatment with the multivitamin preparation. ^f^ Included subsequent sucralfate doses at 5, 10, 15, and 24 h after the moxifloxacin-sucralfate coadministration. ^g^ Included a subsequent ferrous sulfate dose 24 h after the moxifloxacin-ferrous sulfate coadministration. ^h^ Included subsequent calcium doses at 12 and 24 h after the moxifloxacin-calcium coadministration.

**Table 2:** Fluoroquinolone-multivalent cation drug-drug interaction management strategies.

Management Strategy	Considerations
Space out dose administrations	Evidence exists to support this practice in healthy adults but little evidence in those who are ill Allows patient to continue potentially needed drug therapies May not be feasible depending on multivalent cation-fluoroquinolone pair due to the amount of spacing required and/or the amount of administrations per day May add complexity to drug regimen decreasing patients’ likelihood to successfully manage Space out drugs by times that exceed those recommended by manufacturers (Table 3), if possible
Temporarily stop multivalent cation	Assures the multivalent cation will not impact absorption of fluoroquinolone Depending on the indication, could cause patient harm A provider may forget to restart the temporarily-held multivalent cation after the fluoroquinolone course is completed
Select alternative agent for multivalent cation or fluoroquinolone	Assures the multivalent cation will not impact absorption of fluoroquinolone Decreases the use of fluoroquinolones The drug that is temporarily substituted for the multivalent cation may never be discontinued and/or the multivalent cation may never be restarted
Temporarily decrease frequency of multivalent cation	May improve the feasibility of spacing out dose administrations The dose frequency of the multivalent cation may not get adjusted back to its original frequency after completion of the fluoroquinolone
Change fluoroquinolone route from oral to intravenous	Assures the multivalent cation will not impact absorption of fluoroquinolone May delay hospital discharge May decrease patient satisfaction Increases the risk of catheter-associated infections and infusion reactions May increase cost

**Table 3:** Manufacturer-recommendation dose spacing.

Oral Drug	Administration Recommendation Per Prescribing Information
Delafloxacin [23]	Administer delafloxacin at least 2 h before or 6 h after antacids containing magnesium, or aluminum, with sucralfate, with metal cations such as iron, or with multivitamin preparations containing zinc or iron
Moxifloxacin [35]	Administer moxifloxacin tablets at least 4 h before or 8 h after antacids containing aluminum or magnesium, with sucralfate, with metal cations such as iron, or with multivitamins containing iron or zinc
Ciprofloxacin [37] *	Administer ciprofloxacin at least 2 h before or 6 h after magnesium/aluminum antacids; sucralfate; multivitamin preparations with zinc; or other products containing calcium, iron or zinc
Levofloxacin [38] **	Administer levofloxacin at least 2 h before or 2 h after antacids containing magnesium, aluminum, as well as sucralfate, metal cations such as iron, and multivitamin preparations with zinc

* Applies to both the immediate and extended release tablets; also applies to the oral suspension. ** Applies to both the tablet and oral solution formulations.
